# LncRNA GAS5 is upregulated in polycystic ovary syndrome and regulates cell apoptosis and the expression of IL-6

**DOI:** 10.1186/s13048-020-00748-y

**Published:** 2020-12-12

**Authors:** Chunxia Wang, Shishi Yue, Yaru Jiang, Yanjia Mao, Zhijing Zhao, Xinwei Liu, Xiaoqian Zhang, Dongxu Pei, Yongwei Li

**Affiliations:** 1 Department of Reproductive medicine, The Second Affiliated Hospital of Henan, University of TCM, Henan Province 450002 Zhengzhou City, People’s Republic of China; 2 Henan University of TCM, 450046 Zhengzhou City, Henan Province People’s Republic of China; 3 Department of Clinical Laboratory, The Second Affiliated Hospital of Henan University of TCM, No.6 Dongfeng Road, Henan Province 450002 Zhengzhou City, People’s Republic of China

**Keywords:** Polycystic ovary syndrome, GAS5, IL-6, Granulosa, Apoptosis

## Abstract

**Background:**

GAS5 contains a hormone response element that can induce cell apoptosis in breast cancer. It is known that cell apoptosis and hormone response play crucial roles in polycystic ovary syndrome (PCOS), indicating the potential involvement of GAS5 in PCOS. This study was performed to investigate the potential involvement of GAS5 and IL-6 (a critical player in PCOS) in PCOS.

**Methods:**

Research subjects of this study included 60 PCOS patients and 60 healthy controls. The expression levels of GAS5 and IL-6 in plasma of both patients and controls were measured by qPCR and ELISA, respectively. Cell transfections were performed to analyze the interaction between GAS5 and IL-6. Cell apoptosis was analyzed by cell apoptosis assay.

**Results:**

GAS5 was upregulated in plasma of PCOS patients. The expression levels of GAS5 were positively correlated with the expression levels of IL-6. Altered expression levels of GAS5 and IL-6 distinguished PCOS patients from healthy controls. In cells of a granulosa-like tumor cell line (KGN), overexpression of GAS5 led to upregulated IL-6, while silencing of GAS5 played an opposite role. Cell apoptosis analysis showed that overexpression of GAS5 significantly decreased apoptosis rate of KGN cells. Silencing of GAS5 increased the rate of KGN cell apoptosis.

**Conclusions:**

GAS5 is upregulated in PCOS and regulates cell apoptosis and the expression of IL-6.

## Background

Polycystic ovary syndrome (PCOS) is a common type of endocrine and metabolic disorder that affects females at their reproductive age [1]. PCOS patients suffer from excess male hormone production and prolonged or infrequent menstrual periods [2]. The ovary of PCOS patients may not regularly release eggs due to the development of numerous small follicles [3]. Prevalence of PCOS varies across the world. It is generally believed that about 5–20% of women suffer from PCOS before the end of reproductive age [4]. The development of PCOS is accompanied by altered cell behaviors, such as apoptosis, which not only causes infertility [5], but also increases the risk of metabolic disorders and cardiovascular disease [6]. Therefore, effective treatment of PCOS is critical.

Studies on the molecular pathogenesis of PCOS have revealed a considerable number of genetic alterations involved in the pathogenesis of this disease [7, 8]. Functional characterization of genetic players may contribute to the development of novel anti-PCOS therapies [7, 8]. The development of PCOS requires the involvement of non-coding RNAs (ncRNAs) [9], which do not participate in protein synthesis but regulate gene expression at multiple levels to participate in human diseases [10]. GAS5 is a recently characterized tumor suppressive long ncRNA (lncRNA) in multiple types of cancer [11]. It is known that GAS5 can respond to hormone signaling to regulate cell behaviors [12], indicating the potential involvement of GAS5 in PCOS. GAS5 contains a hormone response element that can induce cell apoptosis in breast cancer [12]. It is known that cell apoptosis and hormone response play crucial roles in PCOS [4, 5], indicating the potential involvement of GAS5 in PCOS, which the molecular function of GAS5 in PCOS was uncelar. Our preliminary microarray analysis revealed the upregulation of GAS5 in PCOS and its positively correlation with IL-6, which is a potential player in PCOS [13]. This study firstly was performed to investigate the potential involvement of GAS5 and IL-6 in PCOS.

## Methods

### Research subjects

This study passed the review board of the Ethics Committee of the Second Affiliated Hospital of Henan University of TCM. Research subjects of this study included 60 PCOS patients (20 to 36 years old, mean age 29.1 ± 3.2 years old) as well as 60 healthy controls (20 to 36 years old, mean age 29.0 ± 3.1 years old). These participants were admitted to aforementioned hospital between March 2017 and March 2019. PCOS was diagnosed if a female had: (1) irregular periods; and (2) normal or low follicle stimulating hormone (FSH); and (3) elevated lutenizing hormone (LH); and (4) normal or elevated estrogens (E_2_); and (5) elevated testosterone (TT, >= 7.1 nmol/L in all cases); or (6) cysts in polycystic ovaries revealed by ultrasound exam. All PCOS patients were newly diagnosed cases and no therapies were initiated before this study. Recurrent cases were excluded from this study. All healthy controls were enrolled at the physiological health center of aforementioned hospital after they received systemic physiological examinations during the same time period. The parameters of all physiological functions of all healthy controls were within the normal range. All participants signed the informed consent after they were informed of experiment principle of this study. Key clinical data of the two groups were shown in Table 1.

**Table 1:** Key clinical data of two groups

Variables	PCOS	Control
Number	60	60
Age (years)	29.1 ± 3.2	29.0 ± 3.1
BMI	24.3 ± 1.1*	23.8 ± 0.7
TT (ng/mL)	0.77 ± 0.05*	0.33 ± 0.03
E2 (pg/mL)	47.23 ± 3.77	43.42 ± 3.17
FSH (mIU/mL)	5.77 ± 0.23	6.49 ± 0.42
LH (mIU/mL)	11.48 ± 1.44*	5.13 ± 0.38
LH/FSH	1.99 ± 0.28*	0.79 ± 0.09

*P < 0.05

### Plasma preparation

Under fasting condition, blood (5 ml) was extracted from each patient and the healthy control. Blood samples were transferred to EDTA tubes and centrifuged at 1,200 g at room temperature for 10 min to separate plasma. Plasma samples were stored in a liquid nitrogen sink before the following experiments.

QUICKI and OGTT testQUICKI is determined according to the following formula: QUICKI = 1/[log(I0) + log(G0)], I0 is the fasting insulin, and G0 is the fasting glucose. At the same time, standard OGTT was performed by measurement of blood glucose levels at the baseline and 2-h after oral intake of 75-g glucose.

### Cells and transfections

Following the methods described by Nishi et al. [14], a human granulosa-like tumor cell line (KGN) was established using cells from a PCOS patient. Cell culture medium was composed of 45% DME, 45% F12 and 10% FBS. Cells culture conditions were 37 ºC, 5% CO_2_ and 95% humidity. KGN cells were harvested at confluence of 75–85% to perform the following experiments. GAS5 expression vector was constructed using pcDNA3.1 (Invitrogen) vector as backbone. Negative control (NC) siRNA and GAS5 siRNA were designed and synthesized by Invitrogen. Lipofectamine 2000 (Invitrogen) was used to transfect 10 nM vector or 50 nM siRNA into 10^6^ cells. Cells transfected empty vector or NC siRNA were used as NC cells. Cells without transfections were used as the control (C) cells. Cells were harvested at 24 h post-transfection to perform the following experiments.

### RNA preparation and qPCR assays

Total RNAs were extracted from KGN cells and plasma samples using ISOLATE II RNA Mini Kit (Bioline). All RNA samples were digested with gDNA eraser (Takara) to remove genomic DNA. NanoDrop™ 2000c Spectrophotometer (Thermo Scientific‎) was used to measure RNA concentrations. Tetro Reverse Transcriptase (Bioline) was used to reverse transcribe total RNAs into cDNA. The qPCR reaction mixtures were prepared using LightCycler® 480 SYBR Green I Master (Roche Life Science) to measure the expression levels of GAS5 and IL-6 from both KGN cells and plasma samples with GAPDH as endogenous control. Fold changes of gene expression levels were calculated using 2^−ΔΔCt^ method. All PCR reactions were repeated 3 times. ΔCt = Ct (targeted gene) - Ct (GAPDH). The sample with the biggest ΔCt was set to “1”, all other samples were normalized to this sample.

### ELISA

Human IL-6 ELISA Kit (ELH-IL6, RayBiotech) was used to measure the expression levels of IL-6 in plasma from 60 PCOS patients and the healthy controls. All steps were completed following the instructions of RayBiotech. The expression levels of IL-6 were presented as pg/ml.

### Western-blot

RIPA solution (Invitrogen) was used to extract total proteins from KGN cells at 24 h post-transfection, followed by BCA assay (Invitrogen) to measure protein concentrations. Protein denaturation was performed in boiling water for 10 min. Proteins were separated by performing electrophoresis using 10% SDS-PAGE gel. Gel transfer to PVDF membrane was then performed, followed by blocking in PBS containing 5% non-fat milk at room temperature for 2 h. After that, membranes were incubated with rabbit primary antibodies of GAPDH (ab9485, Abcam) and IL-6 (ab7737, Abcam) at 4 ºC for 18 h, followed by incubation with secondary antibody of anti-rabbit IgG-HRP (ab6721, Abcam) at room temperature for 2 h. ECL Western Blotting Substrate Kit (ab65623, Abcam) was used to produce signals. Quantity One software was used to process data.

### Cell apoptosis assay

Cell apoptosis assay was performed to evaluate the effects of transfections on the apoptosis of KGN cells at 24 h post-transfection. In brief, serum-free single cell suspensions were prepared with a cell density of 10^5^ cells per ml. Cells were cultivated in a 6-well plate (2 ml per well) under aforementioned conditions for 48 h. Cells were then digested with 0.25% trypsin, followed by staining with Annexin V-FITC and propidium iodide (PI) at room temperature for 15 min in dark. Flow cytometry was then performed to detect apoptotic cells.

### Statistical analysis

The sample size provided sufficient statistical power (about 0.90) in all cases. Data of 3 biological replicates involved in each experiment were expressed as mean ± standard deviation values. Differences between 2 groups were explored using an unpaired t test. Differences among multiple groups were explored using Kruskal-Wallis test and post hoc Dunn test. ROC curve analysis was performed for diagnostic analysis. Correlations were analyzed by linear regression. *P* < 0.05 was considered as statistically significant.

## Results

### GAS5 and IL-6 were upregulated in plasma of PCOS patients

**Fig. 1:**
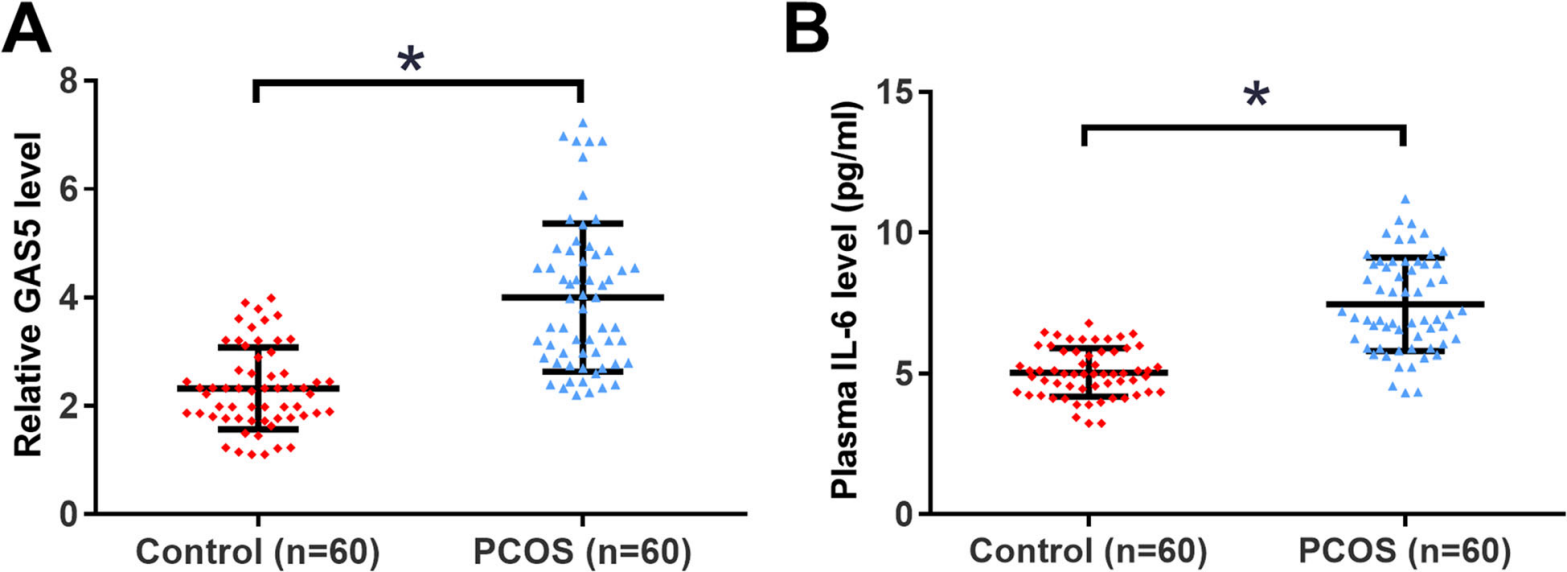
GAS5 and IL-6 were upregulated in plasma of PCOS patients. The differential expression of GAS5 (**a**) and IL-6 (**b**) in PCOS patients was analyzed by performing qPCR and ELISA to measure their levels in plasma from 60 PCOS patients and 60 healthy controls. PCR reactions were repeated 3 times and data were compared by Wilcoxon rank sum test. *, *p* < 0.05

The differential expression of GAS5 and IL-6 in PCOS patients were detected by qPCR and ELISA. Wilcoxon rank sum test showed that the expression levels of GAS5 were significantly higher in plasma of PCOS patients compared to that in the control group (Fig. 1a, *p* < 0.05). Similarly, the expression levels of IL-6 were also significantly higher in PCOS patients than that in the controls (Fig. 1b, *p* < 0.05).

### The expression levels of GAS5 and IL-6 showed diagnostic values for PCOS

**Fig. 2:**
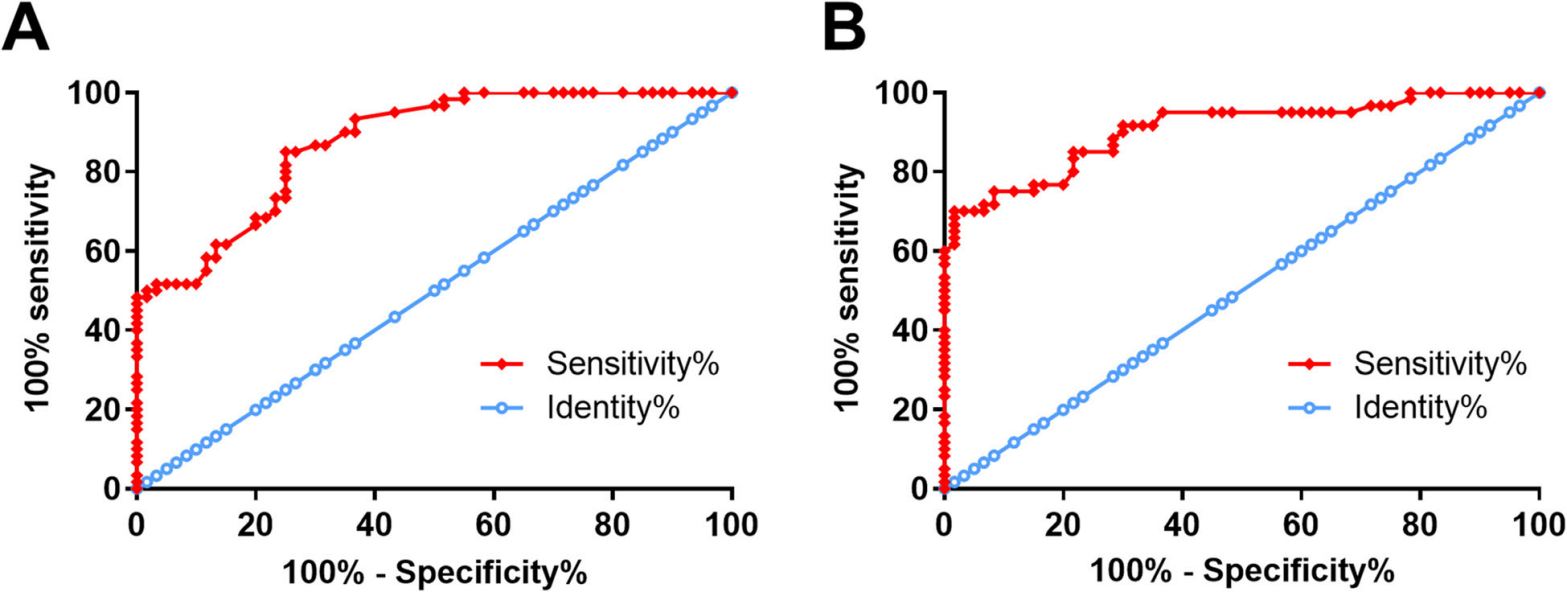
The expression levels of GAS5 and IL-6 showed diagnostic values for PCOS. ROC curve analysis was performed to analyze the diagnostic values of plasma GAS5 (**a**) and IL-6 (**b**) for PCOS. In ROC curve analysis, the true positive cases were PCOS patients and the true negative cases were healthy controls

ROC curve analysis was performed to analyze the diagnostic values of expression levels of GAS5 and IL-6 in plasma for PCOS. In ROC curve analysis, the true positive cases were PCOS patients and the true negative cases were healthy controls. For plasma GAS5, area under the curve (AUC) was 0.87 (95% confidence interval: 0.82–0.93; standard error: 0.030) (Fig. 2a). For plasma IL-6, AUC was 0.91 (95% confidence interval: 0.85–0.96; standard error: 0.027) (Fig. 2b).

### The relationship between GAS5 and IL-6, insulin levels, blood glucose levels were evaluated in PCOS patients

**Fig. 3:**
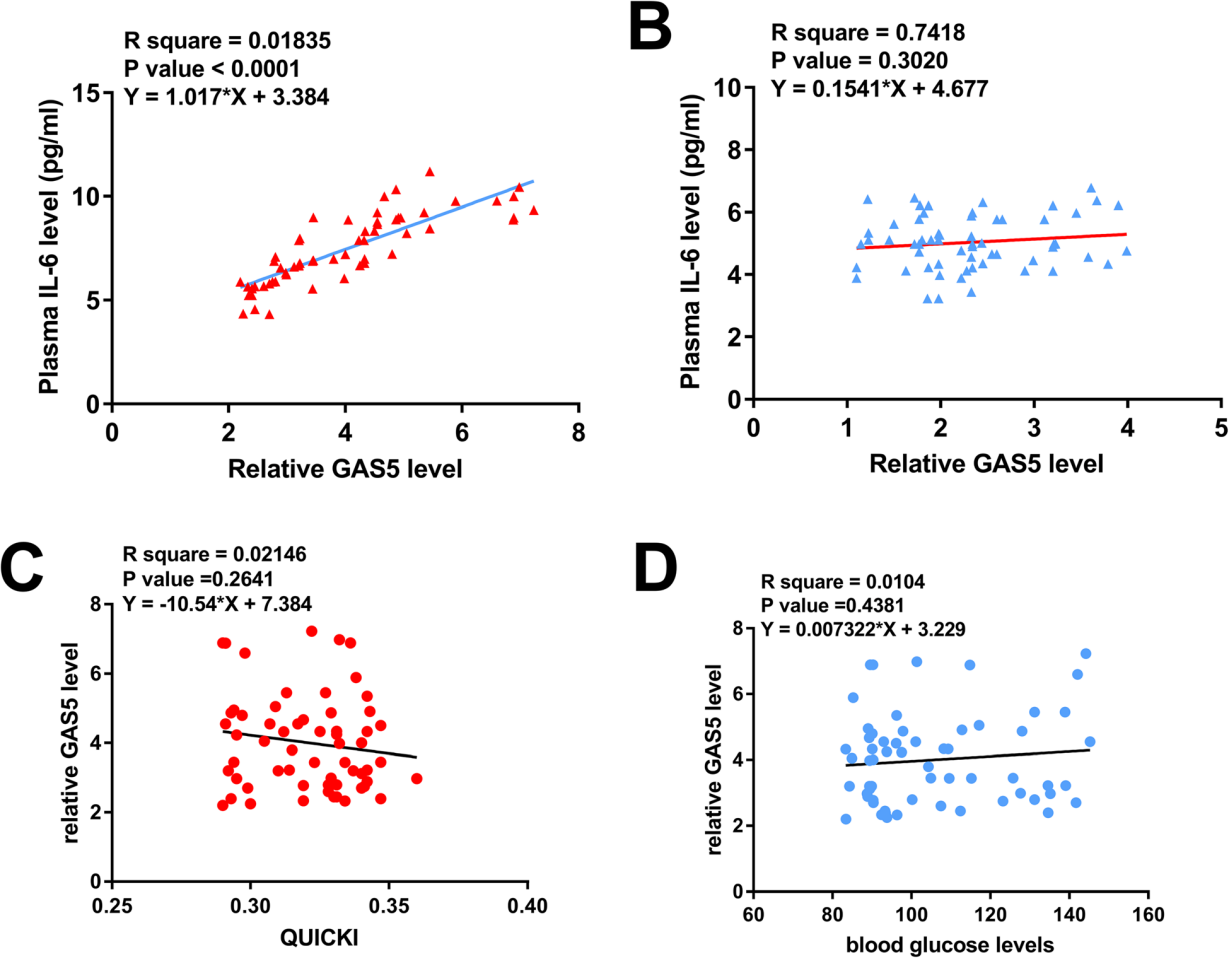
The relationship between GAS5 and IL-6, insulin levels, blood glucose levels were evaluate in PCOS patients. The correlations between plasma levels of GAS5 and IL-6 across PCOS samples (**a**) and control samples (**b**) were analyzed by linear regression. The relationship between GAS5 and insulin levels(**c**), blood glucose levels(**d**) were evaluate in PCOS patients

The correlations between the expression levels of GAS5 and IL-6 were analyzed by linear regression. It was observed that the expression levels of GAS5 were significantly and positively correlated with the expression levels of IL-6 across PCOS samples (Fig. 3a). However, no significant correlation was observed between the expression levels of GAS5 and IL-6 across the control samples (Fig. 3b). It is worth noting that QUICKI in PCOS patients ranged from 0.29 to 0.34, with a mean of 0.32 ± 0.03. Linear regression analysis revealed a slight inverse correlation between the expression levels of QUICKI and GAS5 (Fig. 3c, *p* > 0.05). OGTT data of all PCOS patients were collected, but we observed no significant correlation between GAS5 and blood glucose levels by linear regression analysis(Fig. 3d, *p* > 0.05).

### GAS5 positively regulated IL-6 in KGN

**Fig. 4:**
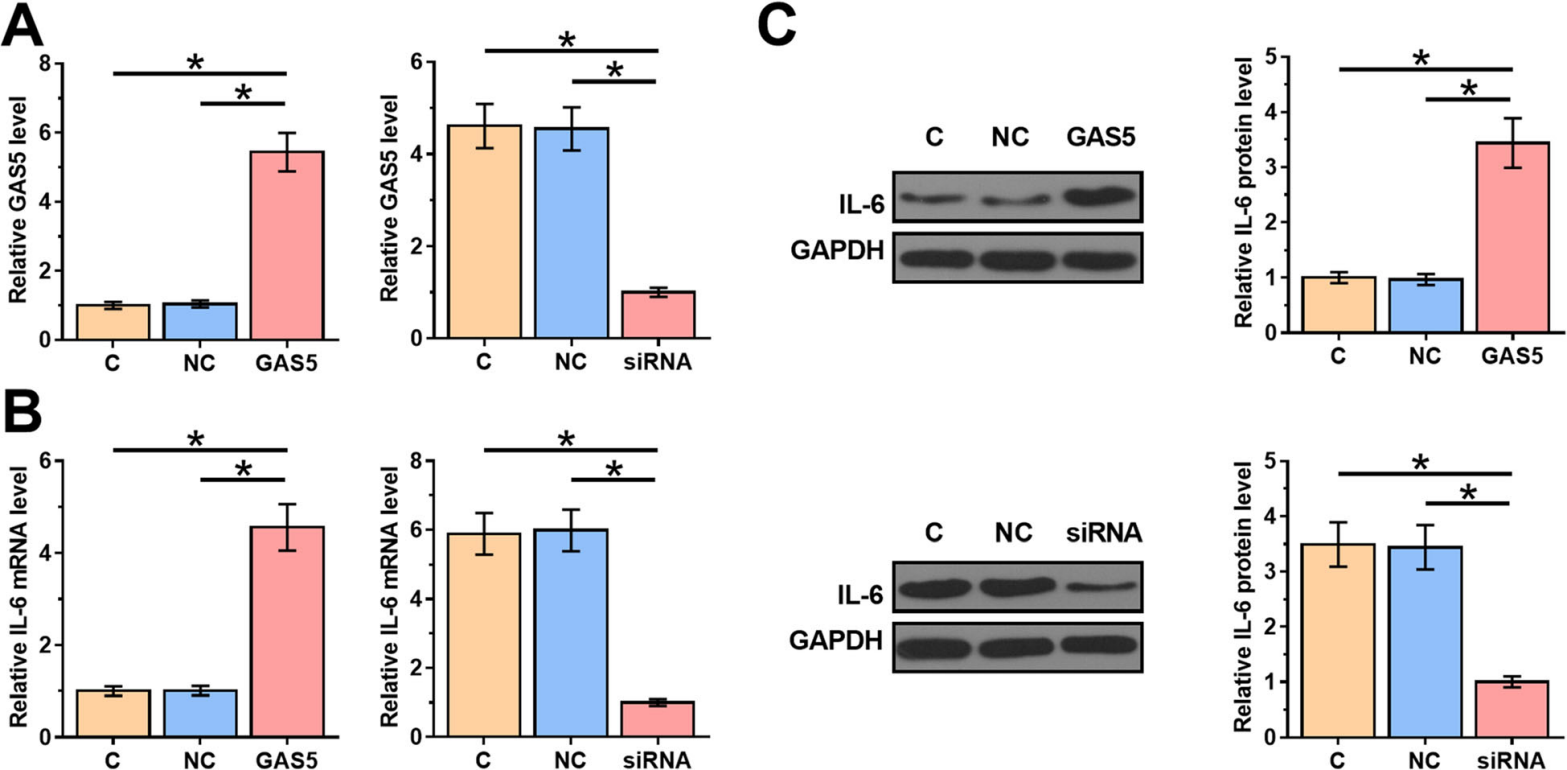
GAS5 positively regulated IL-6 in KGN. KGN cells were transfected with GAS5 expression vector or GAS5 siRNA to analyze the relationship between GAS5 and IL-6. Transfections were confirmed by qPCR at 24 h post-transfection (**a**). The effects of overexpression and silencing of GAS5 on the expression of IL-6 in KGN cells were analyzed by qPCR and western blot at mRNA (**b**) and protein (**c**) level, respectively. Experiments were repeated 3 times and mean values were presented. *, *p* < 0.05

KGN cells were transfected with GAS5 expression vector or GAS5 siRNA to analyze the relationship between GAS5 and IL-6. Transfections were confirmed by qPCR at 24 h post-transfection (Fig. 4a, *p* < 0.05). The effects of overexpression and silencing of GAS5 on the expression of IL-6 in KGN cells were analyzed by qPCR and western blot at mRNA (Fig. 4b) and protein (Fig. 4c) level, respectively. Compared with C and NC groups, overexpression of GAS5 led to promoted expression levels of IL-6, while silencing of GAS5 played an opposite role (*p* < 0.05).

### GAS5 and testosterone negatively regulated KGN cell apoptosis and testosterone upregulated the levels of GAS5

**Fig. 5:**
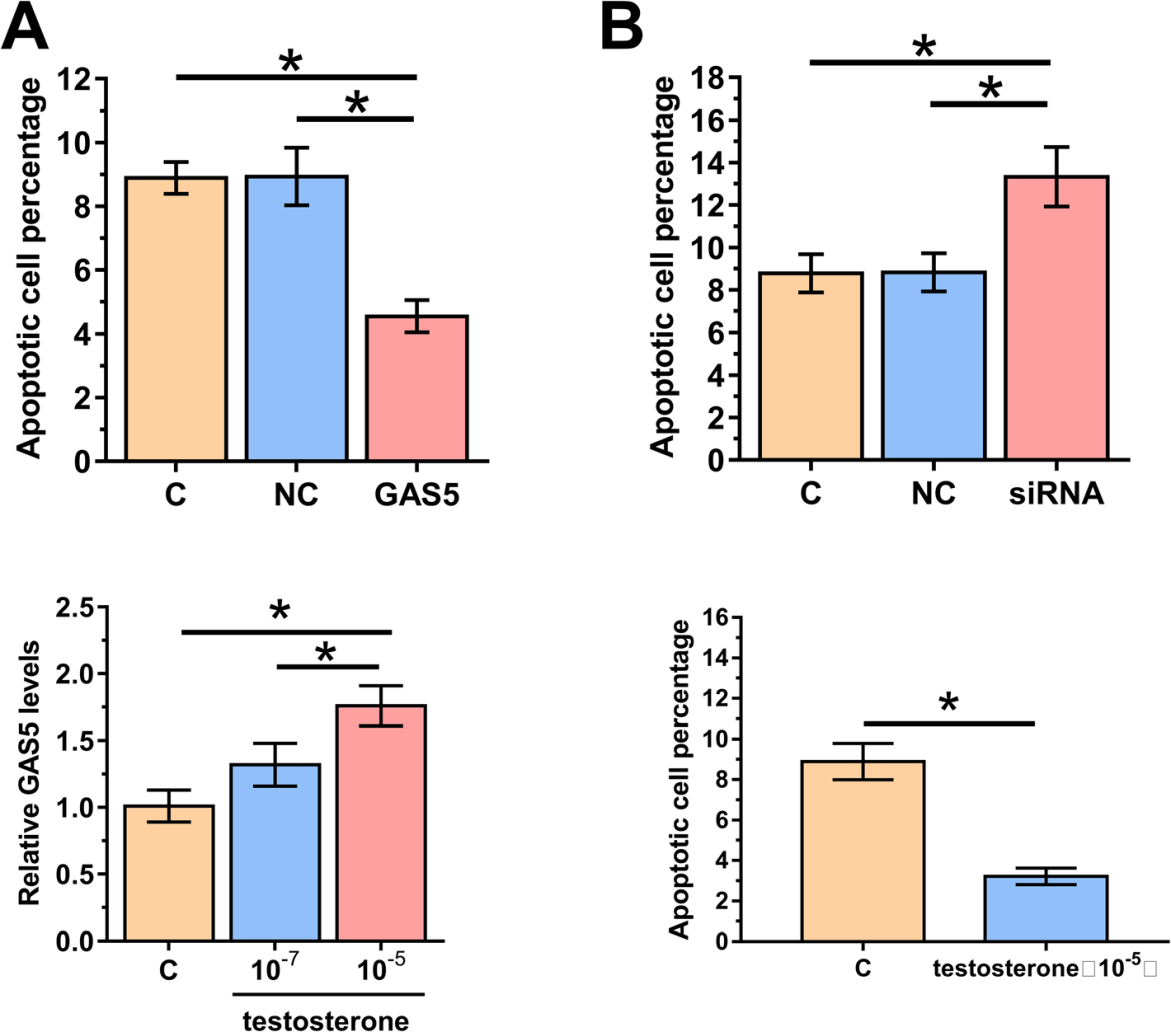
GAS5 and testosterone negatively regulated KGN cell apoptosis and testosterone upregulated the levels of GAS5. The effects of overexpression (**a**) and silencing (**b**) of GAS5 on the apoptosis of KGN cells were analyzed by cell apoptosis assay. KNG were treated with 0.1 nM or 10 nM testosterone for 24 h, followed by detection of GAS5 expression(**c**). Cell apoptosis analysis after KNG were treated with 10 nM testosterone (**d**). Experiments were repeated 3 times and mean values were presented. *, *p* < 0.05

The effects of overexpression and silencing of GAS5 on the apoptosis of KGN cells were assessed by cell apoptosis assay. Compared with C and NC groups, overexpression of GAS5 decreased the rate of KGN cell apoptosis (Fig. 5a, *p* < 0.05). In contrast, silencing of GAS5 increased the rate of KGN cell apoptosis (Fig. 5b, *p* < 0.05). Moreover, since PCOS is frequently characterized by hyperandrogenemia [15], we supposed that the expression difference of GAS5 in our findings was increased by the increase of testosterone. We explored the effect of testosterone on the expression level of GAS5 by qRT-PCR treated with 0.1 nM or 10 nM testosterone for 24 h. GAS5 expression was gradually improved (Fig. 5c, *p* < 0.05). Simultaneously, the apoptotic rate of KGN was significantly decreased after testosterone treatment (Fig. 5d, *p* < 0.05).

## Discussion

This study mainly investigated the involvement of GAS5 in PCOS. We found that GAS5 was upregulated in PCOS and may regulate cell prognosis and IL-6 production to participate in this disease.

The expression pattern and functions of GAS5 have only been investigated in cancer biology [16, 17]. GAS5 is downregulated in different types of cancer and can regulate cancer cell behaviors, such as promoting cancer cell apoptosis and inducing cell proliferation, invasion and migration to promote cancer progression [16, 17]. This study is the first to report the upregulation of GAS5 in PCOS patients. In addition, overexpression of GAS5 led to reduced apoptotic rate of KGN cells. Therefore, GAS5 may regulate cell apoptosis to participate in PCOS. Our data and previous studies showed that GAS5 may play opposite roles in cell apoptosis in different types of disease. It is known that GAS5 can respond to hormone signaling to regulate cell apoptosis [12]. PCOS is characterized by the altered production of hormones [1] Therefore, GAS5 may also interact with hormone signaling to regulate KGN cell behaviors.

The development of PCOS is accompanied by chronic inflammation, which is characterized by the elevated production of pro-inflammatory factors, such as IL-6 [13, 18]. Consistent with previous studies, our study also showed the upregulation of IL-6 in PCOS patients. It has been reported that the expression of IL-6 can be regulated by certain lncRNAs [19]. In this study we showed that GAS5 can positively regulate the expression of IL-6 in KGN cells. However, the mechanism is still unclear. Our correlation analysis showed that the expression levels of GAS5 and IL-6 were positively correlated with each other only in PCOS patients but not in the healthy controls. Therefore, the interaction between GAS5 and IL-6 may be regulated by certain pathological mediators. Of note, PCOS is characterized by hyperandrogenism, which leads to PCO morphology and ovulatory dysfunction in women with PCOS [20]. Our study demonstrated that testosterone inhibited apoptosis possibly by repressing GAS5.

It has been well established that insulin-resistance plays a critical role in the pathogenesis of PCOS. In the past decade, sufficient in vitro and in vivo evidence has supported the pivotal role of insulin resistance and compensatory hyperinsulinemia in the pathogenesis of PCOS [21]. In effect, with the advantage of effectiveness and high safety, insulin-sensitizers are promising medicines to treat PCOS [21–23]. Therefore, our future studies may focus on the involvement of GAS5 in the regulation of insulin-resistance in PCOS. Molecular pathways, such as apoptotic pathways are critical regulators in gynecological conditions, such as endometriosis. In this study, we were not able to characterize pathways that mediated the role of GAS5 in regulating cell apoptosis [24–26]. Our future studies will also explore the potential crosstalk between GAS5 and these molecular pathways, especially apoptotic pathways in gynecological conditions.

Our study is the first to report the alteration of GAS5 in PCOS as well as it role in regulating KGN cell apoptosis and the expression of IL-6. Our study suggested that GAS5 could serve as a potential target for the treatment of PCOS. However, our study is limited by the small sample size. In addition, animal model experiments are also needed to further confirm the function of GAS5 in PCOS *in vivo*.

## Conclusions

In conclusion, GAS5 is upregulated in PCOS and can participate in this disease by regulating cell apoptosis and IL-6 expression.

## Data Availability

The datasets generated and/or analyzed during the current study are not publicly available due research design, but are available from the corresponding author on reasonable request.

## Abbreviations

PCOS: Polycystic ovary syndrome
KGN: Granulosa-like tumor cell line
ncRNAs: Non-coding RNAs
lncRNA: Long ncRNA
AUC: Area under the curve
